# Potenzielle Auswirkungen erhöhter Alkoholsteuern auf die alkoholbedingte Krankheitslast in Deutschland: Eine Modellierungsstudie

**DOI:** 10.1007/s00103-022-03528-9

**Published:** 2022-04-19

**Authors:** Carolin Kilian, Pol Rovira, Maria Neufeld, Jakob Manthey, Jürgen Rehm

**Affiliations:** 1 Institut für Klinische Psychologie und Psychotherapie, Technische Universität Dresden, Chemnitzer Str. 46, 01187 Dresden, Deutschland; 2 Program on Substance Abuse, Public Health Agency of Catalonia, Barcelona, Spanien; 3 WHO European Office for Prevention and Control of Noncommunicable Diseases, Moskau, Russland; 4 Zentrum für Interdisziplinäre Suchtforschung (ZIS), Klinik für Psychiatrie und Psychotherapie, Universitätsklinikum Hamburg-Eppendorf (UKE), Hamburg, Deutschland; 5 Klinik und Poliklinik für Psychiatrie und Psychotherapie, Universitätsklinikum Leipzig, Leipzig, Deutschland; 6 Institute for Mental Health Policy Research, Centre for Addiction and Mental Health, Toronto, Kanada; 7 Dalla Lana School of Public Health, University of Toronto, Toronto, Kanada; 8 Department of Psychiatry, University of Toronto, Toronto, Kanada; 9 Department of International Health Projects, Institute for Leadership and Health Management, I.M. Sechenov First Moscow State Medical University, Moskau, Russland

**Keywords:** Alkoholkonsum, Spezifische Steuern auf alkoholische Getränke, Vermeidbare Krankheitsfälle, Alkoholbedingte Krankheitsfälle, Alkoholpolitik, Alcohol consumption, Excise taxes on alcoholic beverages, Avoidable diseases, Alcohol-attributable diseases, Alcohol policy

## Abstract

**Hintergrund:**

Deutschland gehörte im Jahr 2019 zu den Ländern mit dem weltweit höchsten Pro-Kopf-Alkoholkonsum, welcher wesentlich zur Krankheitslast beiträgt.

**Fragestellung:**

In dieser Modellierungsstudie schätzen wir, wie viele alkoholbedingte inzidente Krankheits- sowie Todesfälle in Deutschland im Jahr 2019 hätten vermieden werden können, wenn die derzeitigen Verbrauchssteuern auf Alkohol um 20 %, 50 % und 100 % erhöht worden wären.

**Methodik:**

Ausgangspunkt für die Modellierung sind die spezifischen Verbrauchssteuern auf alkoholische Getränke in Deutschland. Drei Szenarien wurden unter der Annahme, dass die resultierende Steuererhöhung vollständig in den Verkaufspreis übertragen wird, und unter Verwendung von getränkespezifischen Preiselastizitäten modelliert. Mittels des sich daraus ergebenden Rückgangs im jährlichen Pro-Kopf-Konsum und der krankheitsspezifischen Risikofunktionen wurde die vermeidbare alkoholbedingte Inzidenz bzw. Mortalität geschätzt. Berücksichtigt wurden alkoholbedingte Erkrankungen des Herz-Kreislauf- und Verdauungssystems, Alkoholabhängigkeit, Epilepsie, Infektionskrankheiten sowie Verletzungen und Unfälle.

**Ergebnisse:**

Insgesamt hätten durch eine Verdoppelung der spezifischen Verbrauchssteuern auf Alkohol im Jahr 2019 bis zu 200.400 alkoholbedingte Erkrankungs- und Verletzungsfälle sowie 2800 Todesfälle vermieden werden können. Dies entspricht knapp 7 % der berücksichtigten alkoholbedingten Krankheits- bzw. Todesfälle in Deutschland.

**Diskussion:**

Alkoholbedingte Erkrankungen und Verletzungen sind vermeidbar und eine Erhöhung der spezifischen Verbrauchssteuern auf alkoholische Getränke in Deutschland könnte die alkoholbedingte Krankheitslast substanziell reduzieren.

**Zusatzmaterial online:**

Zusätzliche Informationen sind in der Online-Version dieses Artikels (10.1007/s00103-022-03528-9) enthalten.

## Abstract (en)

**Background:**

In 2019, Germany was among the countries with the highest alcohol per capita consumption in the world, which contributes significantly to the burden of disease.

**Aim:**

In this modelling study, we estimate how many alcohol-attributable diseases and deaths in Germany could have been avoided in 2019 if current alcohol excise taxes were increased by 20%, 50%, and 100%.

**Methods:**

The starting point for the modelling was the national beverage-specific alcohol taxes. Three scenarios were modelled under the assumption that the resulting tax increase would be fully transferred to the retail prices. Beverage-specific price elasticities were used. Based on the estimated resulting decline in annual per capita consumption and the disease-specific risk functions, we modelled the avoidable incidence and mortality for alcohol-attributable diseases for 2019. Alcohol-attributable diseases of the cardiovascular and digestive systems, alcohol dependence, epilepsy, and infectious diseases as well as injuries and accidents were considered.

**Results:**

Overall, doubling the beverage-specific alcohol taxes could have avoided up to 200,400 alcohol-attributable cases of disease and injury as well as 2800 deaths in Germany in 2019. This corresponds to just under 7% of the modelled new alcohol-attributable cases of disease and death in Germany.

**Discussion:**

Alcohol-attributable diseases and injuries are preventable and an increase in the alcohol taxes could substantially reduce the alcohol-attributable burden of disease in Germany.

## Einleitung

Deutschland zählte im Jahr 2019 zu den Ländern mit dem weltweit höchsten Pro-Kopf-Alkoholkonsum [1]. Alkoholkonsum ist ein entscheidender und veränderbarer Risikofaktor für eine Vielzahl von Erkrankungen, einschließlich verschiedener Krebs- und kardiovaskulärer Erkrankungen, die einen bedeutsamen Anteil der gesamten Krankheitslast in Deutschland ausmachen [2, 3]. Die Eigenschaft der Veränderbarkeit des Risikofaktors bedeutet, dass die alkoholbedingte Krankheitslast vermeidbar wäre, wenn kein oder weniger Alkohol getrunken würde. Erst kürzlich schätzte eine Modellierungsstudie, dass über einen 30-Jahres-Zeitraum ca. 244.000 alkoholbedingte Krebserkrankungen in Deutschland vermieden werden könnten, wenn die Empfehlungen eines weniger riskanten Konsums (Frauen: < 10 g Reinalkohol pro Tag, Männer: < 20 g Reinalkohol pro Tag) eingehalten würden [4]. Eine Reduzierung des Alkoholkonsums auf gesamtgesellschaftlicher Ebene ist ein explizites Ziel in mehreren internationalen Vereinbarungen, wie beispielsweise dem Globalen Aktionsplan zur Prävention und Kontrolle von nichtübertragbaren Krankheiten 2013–2020 der Weltgesundheitsorganisation (WHO; [5]) sowie den nachhaltigen Entwicklungszielen der Vereinten Nationen (Ziel 3.5 [6]).

Zur Reduzierung der alkoholbedingten Krankheitslast schlägt die WHO verschiedene, evidenzbasierte alkoholpolitische Maßnahmen vor und hebt dabei besonders kosteneffektive und praktikable Maßnahmen (sog. Best Buys) hervor [7]. Diese umfassen die Erhöhung der alkoholspezifischen Verbrauchssteuern, Einschränkungen in der Verfügbarkeit alkoholischer Getränke sowie Einschränkungen oder Verbote von Alkoholwerbung und -marketing [8, 9]. Trotz der umfassenden Studienlage, die den gesundheitlichen Nutzen durch höhere alkoholspezifische Verbrauchssteuern aufzeigt (siehe z. B. [10–12]), sind die aktuellen Verkaufspreise für alkoholische Getränke in der Europäischen Union (EU) und insbesondere in Deutschland sehr niedrig [13]. Die zugrunde liegende Steuerstruktur sowie eine Mindeststeuer auf alkoholische Getränke ist durch die EU gesetzlich geregelt, mit dem Ziel die Verbrauchssteuern auf alkoholische Getränke zwischen den Mitgliedstaaten zu harmonisieren [14].

In Deutschland gibt es insgesamt vier Verbrauchssteuern, die die Besteuerung verschiedener alkoholischer Getränke regeln:

1. Die *Alkoholsteuer* definiert die zu entrichtende Steuer auf Ethylalkohol in Spirituosen und anderen alkoholhaltigen Waren und basiert auf dem Alkoholgehalt des zu versteuernden Produkts [15].
2. Die *Biersteuer* legt die Verbrauchssteuer von Bier fest, wobei sich die zu entrichtende Steuer durch die Stammwürze und folglich den Alkoholgehalt ergibt [16].
3. Die Verbrauchssteuer für *Schaumweine* sowie Zwischenerzeugnisse richtet sich nach dem Gesamtvolumen des fertigen Produkts [17]. Eine Verbrauchssteuer auf *stillen Wein* gibt es in Deutschland nicht.
4. Über die Erhebung einer *Sondersteuer auf alkoholhaltige Süßgetränke* (Alkopops) definiert das Gesetz eine weitere Verbrauchssteuer [18].

Im europäischen Vergleich hat Deutschland eine der geringsten Verbrauchssteuern auf alkoholische Getränke [19], wodurch Alkohol hierzulande besonders erschwinglich ist [20].

In dieser Publikation schätzen wir, wie sich erhöhte Verbrauchssteuern auf alkoholische Getränke auf die alkoholbedingte Krankheitslast in Deutschland auswirken würden. Dabei vergleichen wir die beobachtete alkoholbedingte Krankheitslast im Jahr 2019 mit 3 hypothetischen Szenarien, in denen die derzeitigen getränkespezifischen Verbrauchssteuern um 20 %, 50 % bzw. 100 % angehoben würden.

## Methodik

Die Methode dieser Modellierungsstudie basiert im Wesentlichen auf einer Erweiterung der Studie von Rovira et al. [21]. Nachfolgend werden die wichtigsten Schritte und Annahmen der vorliegenden Arbeit dargestellt.

### Alkoholbedingte Erkrankungen und Unfälle

Tab. 1 gibt eine Übersicht über die Erkrankungen und Verletzungen, die in diese Studie einbezogen wurden. Dabei werden solche Diagnosen berücksichtigt, die durch Alkoholkonsum verursacht werden und kurzfristig, das heißt innerhalb eines Jahres, auftreten können (siehe [22]). Folgende alkoholbedingte Krankheitsdiagnosen wurden nicht oder nur teilweise berücksichtigt:

1. Nicht modelliert wurden alkoholbedingte Krebserkrankungen (Diagnosen entsprechend der 10. Version der Internationalen statistischen Klassifikation der Krankheiten und verwandter Gesundheitsprobleme, ICD-10: C00–C08, C09–C10, C12–C14, C15, C18–C21, C22, C32, C50), da infolge einer Erhöhung der derzeitigen Verbrauchssteuern auf alkoholische Getränke keine kurzfristigen Veränderungen in der Krankheitsinzidenz bzw. Mortalität zu erwarten sind. Grund hierfür ist eine mittlere Latenzzeit von 10 Jahren zwischen Exposition (Alkoholkonsum) und Krebsentwicklung [23].
2. Da die Wirkung von Alkohol (sowohl protektiv als auch krankheitsfördernd) auf die Inzidenz von ischämischen Herzkrankheiten (ICD-10: I20–25) schwer zu quantifizieren und umstritten ist [24], wird diese Krankheitsgruppe nicht berücksichtigt.
3. Für Infektionskrankheiten (humane Immundefizienzviruskrankheit (HIV), ICD-10: B20–B24; Tuberkulose, ICD-10: A15–A19, B90; sowie für Infekte der unteren Atemwege, ICD-10: J09–22, P23, U04) wurden ausschließlich vermeidbare Krankheitsfälle, jedoch keine vermeidbaren Todesfälle modelliert. Diese Entscheidung wurde getroffen, da sich der unmittelbare Einfluss von Änderungen im Alkoholkonsum bei der Entstehung von Infektionskrankheiten besser quantifizieren lässt als für Todesfälle [25].
4. Es standen keine Daten zur Inzidenz von Hypertonie zur Verfügung, weshalb ausschließlich die vermeidbare alkoholbedingte Mortalität geschätzt wurde.

**Table 1:** 

Erkrankung und Verletzungen	ICD-10-Code
*Infektionskrankheiten*^a^	
Tuberkulose	A15–19, B90
Humane Immundefizienzviruskrankheit	B20–24
Infekte der unteren Atemwege	J09–22, P23, U04
*Erkrankungen des Herz-Kreislauf-Systems*	
Ischämischer Hirninfarkt	G45–46.8, I63–63.9, I65–66.9, I67.2–67.848, I69.3–69.4
Hämorrhagischer Schlaganfall	I60–62.9, I67.0–67.1, I69.0–69.298
Hypertonie^b^	I10–I15
100 % AB kardiovaskuläre Erkrankungen (Endokarditis, Kardiomyopathie und Myokarditis)	I30–I33, I40, I42, I38
*Erkrankungen des Verdauungssystems*	
Leberzirrhose	K70, K74
Pankreatitis	K85–86
*Alkoholabhängigkeit *(100 % AB)	F10, G72.1, Q86.0, X45
*Epilepsie*	G40–G41
*Unfälle und Verletzungen*	
Transportmittelunfälle	V01–04, V06, V09–80, V87, V89, V99
Nicht intentionale Verletzungen	V01–X40, X43, X46–59, Y40–86, Y88, Y89 (ausgenommen sind Transportmittelunfälle)
Vorsätzliche Selbstbeschädigung	X60–84, Y870

100 % AB: Erkrankungen sind zu 100 % alkoholbedingt, d. h. vollständig auf Alkohol zurückführbar

^a^ Für Infektionskrankheiten wurden ausschließlich vermeidbare, alkoholbedingte Krankheitsfälle modelliert

^b^ Für Hypertonie wurde ausschließlich vermeidbare, alkoholbedingte Todesfälle modelliert

Daten zur Krankheitsinzidenz sowie Mortalität (Alter 15+) wurden für das Jahr 2019 der *Global-Burden-of-Disease-*Studie entnommen [26].

### Bestimmung des Referenzszenarios

Als Referenzszenario bezeichnen wir nachfolgend die Besteuerung alkoholischer Getränke im Jahr 2020, also den Anteil der getränkespezifischen Verbrauchssteuern am mittleren Verkaufspreis. Hierfür wurden zunächst die Informationen zu den Verbrauchssteuersätzen sowie der durchschnittlichen Verkaufspreise für 2020 identifiziert. Da weniger als 1 % des registrierten Gesamtalkoholkonsums in Deutschland in Form von anderen Getränken als Bier, Wein und Spirituosen konsumiert wird [27], nahmen wir die Modellierung nur für Bier, Wein und Spirituosen vor. Die Verbrauchssteuer auf Bier beträgt 0,787 € je Grad Plato (Maßeinheit der Stammwürze) pro Hektoliter Bier und für Ethylalkohol (Spirituosen) 1303,00 € je Hektoliter Reinalkohol [14]. Für Bier wurde eine Stammwürze von 12° Plato angenommen und als durchschnittlicher Alkoholgehalt wurde 35 % für Spirituosen gewählt (siehe [28]). Die folgenden durchschnittlichen Verkaufspreise für 2020 wurden ermittelt [13]: Bier (2,43 € je Liter), Wein (7,01 € je Liter) und Spirituosen (16,64 € je Liter). Gemäß den Verbraucherpreisindices haben sich die Verkaufspreise zwischen 2019 und 2020 nur geringfügig verändert [29], weshalb die Verwendung der Daten aus 2020 für das Jahr 2019 zulässig ist. Die folgenden Anteile der Verbrauchssteuern am Verkaufspreis wurden für das Referenzszenario ermittelt: 3,9 % für Bier sowie 31,3 % für Spirituosen. Um trotz fehlender Verbrauchssteuer auf stillen Wein den Einfluss einer hypothetischen Verbrauchssteuererhöhung für Weine zu modellieren, wurde für stillen Wein der gleiche Anteil der Verbrauchssteuer am Produktpreis angenommen wie für Bier (Anteil der Verbrauchssteuer am Verkaufspreis: 3,9 %). Schaumweine, die in Deutschland einer gesonderten Verbrauchssteuer unterliegen [14], wurden nicht berücksichtigt, da der Anteil von Schaumweinen am Pro-Kopf-Weinverbrauch im Jahr 2019 verhältnismäßig gering war (15 %; entspricht weniger als 5 % des Gesamt-Pro-Kopf-Alkoholkonsums [30]).

### Schätzung der Auswirkung erhöhter Verbrauchssteuern auf den Alkoholkonsum

Zunächst wurde die Auswirkung erhöhter getränkespezifischer Verbrauchssteuern auf das Kauf- und damit Trinkverhalten der Konsumierenden geschätzt [31]. Hierfür wurde angenommen, dass die Steuererhöhung vollständig auf den Verkaufspreis und damit auf die Konsumierenden übertragen würde. In einer Sensitivitätsanalyse modellierten wir außerdem ein konservatives Alternativszenario, in dem nur 80 % der Steuererhöhung über den Verkaufspreis auf die Konsumierenden übertragen würde. Veränderungen des Kaufverhaltens durch die Erhöhung des Verkaufspreises werden mittels Preiselastizitäten bestimmt, die Veränderungen im Kaufverhalten bei einer Verdopplung des Verkaufspreises beschreiben. Die Preiselastizität eines Produkts ist dabei von der Getränkepräferenz abhängig, wobei die Preiselastizität für bevorzugte Produkte geringer ist [32, 33]. Entsprechend der Anteile des Bier‑, Wein- bzw. Spirituosenkonsums am jährlichen Pro-Kopf-Konsum von Alkohol in Deutschland [1] wurden die folgenden Werte genutzt: −0,36 (95 % Konfidenzintervall [KI]: −0,48, −0,24) für Bier, −0,60 (95 % KI: −0,72, −0,48) für Wein sowie −1,20 (95 % KI: −1,44, −0,96) für Spirituosen (basierend auf [32, 33]). Da die Impulskontrolle hinsichtlich der Menge des Alkoholkonsums bei Personen mit hohem Alkoholkonsum oder Alkoholabhängigkeit eingeschränkt sein kann (zur Definition: [34]; Überblick der empirischen Studien zur Preiselastizität: [35]), wurde für Personen mit besonders hohem Alkoholkonsum (Frauen: > 40 g Reinalkohol pro Tag, Männer: > 60 g Reinalkohol pro Tag) eine getränketypunabhängige, niedrigere Preiselastizität von −0,28 (95 % KI: −0,37, −0,19) berücksichtigt [35].

Expositionsdaten zum Alkoholkonsum in Deutschland wurden für das Jahr 2019 aus einer internationalen Modellierungsstudie entnommen (durchschnittlicher registrierter Pro-Kopf-Konsum für 2019: 10,9 l Reinalkohol; [1]). Auf Empfehlung der WHO *Technical Advisory Group on Alcohol and Drug Epidemiology* wurden, wie in den meisten solcher Analysen (z. B. [36]), nur 80 % des Pro-Kopf-Konsums verwendet, um für nicht getrunkenen Alkohol (z. B. Restbestände in Flaschen, zerbrochene Flaschen und verschütteten Alkohol) sowie für die Unterschätzung des Alkoholkonsums in medizinisch-epidemiologischen Studien zur Risikoermittlung zu korrigieren [37].

### Schätzung vermeidbarer alkoholbedingter Krankheits- und Todesfälle

Anschließend wurde der Effekt des reduzierten Alkoholkonsums für die 3 Szenarien der Verbrauchssteuererhöhung auf die Inzidenz bzw. Mortalität der in Tab. 1 gelisteten alkoholbedingten Erkrankungen und Verletzungen berechnet. Hierfür wurden die alkoholattributablen Fraktionen je Erkrankung bzw. Verletzung nach Alter und Geschlecht für jedes Szenario mittels der geschlechter- und krankheitsspezifischen Risikofunktion berechnet und mit den Inzidenzen bzw. Mortalitätsfällen multipliziert. Die daraus resultierenden alkoholattributablen Inzidenzen bzw. Mortalitätsfälle wurden anschließend mit dem Referenzszenario verglichen.

Es wurden 2 Sonderfälle in der Modellierung berücksichtigt:

1. Neben dem Gesamtalkoholkonsum sind alkoholbedingte Verletzungen und Unfälle mit Rauschtrinkepisoden (Trinkgelegenheiten, in denen mehr als 60 g Reinalkohol konsumiert werden) assoziiert [2, 36]. Aus diesem Grund wurde zur Schätzung vermeidbarer alkoholbedingter Verletzungen und Unfälle nicht nur der Pro-Kopf-Konsum, sondern ebenfalls die Prävalenz von Rauschtrinkepisoden einbezogen. Hierfür wurde angenommen, dass nach Erhöhung der Verbrauchssteuern auf alkoholische Getränke die Prävalenz von Rauschtrinkepisoden proportional zum Pro-Kopf-Konsum zurückgeht.
2. Die alkoholattributable Fraktion für Erkrankungen, die vollständig auf den Konsum von Alkohol zurückzuführen sind, beträgt unabhängig vom Modellierungsszenario stets 100 %. Die Anzahl vermeidbarer Erkrankungsfälle bzw. Todesfälle wurde deshalb über die Differenz in den absoluten Krankheits- bzw. Todesfällen je Szenario geschätzt (basierend auf [38]). Eine detaillierte Beschreibung der Modellierung ist im Onlinematerial 1 verfügbar.

Die Risikofunktionen, die den krankheitsspezifischen Zusammenhang zwischen Exposition und Erkrankung bzw. Verletzung beschreiben, stammen aus einer Publikation von Shield et al. [36]. Konfidenzintervalle wurden mittels Monte-Carlo-Simulation (1000 Iterationen) geschätzt [39].

## Ergebnisse

Unter Berücksichtigung der in die Modellierung einbezogenen Diagnosen gab es im Jahr 2019 10,9 Mio. (95 % KI: 10.574.732–11.274.436) inzidente Krankheitsfälle sowie knapp 174.903 (95 % KI: 166.422–183.390) Todesfälle in Deutschland, bei denen der Konsum von Alkohol eine mögliche Ursache darstellte (alkoholbezogene Krankheits- bzw. Todesfälle). Dies entspricht ca. 3,0 % aller inzidenten Krankheitsfälle bzw. 18,3 % aller Todesfälle. Dabei ist zu beachten, dass in unserer Modellierungsstudie nicht alle Erkrankungen berücksichtigt wurden, die mit Alkohol in Bezug stehen. Circa ein Viertel dieser alkoholbezogenen Krankheits- und Todesfälle waren auf den Konsum von Alkohol zurückzuführen (alkoholbedingte Inzidenz: 2.906.428, 95 % KI: 2.228.037–3.537.265; alkoholbedingte Mortalität: 41.235, 95 % KI: 38.125–45.085). Unfälle und Verletzungen machten dabei mit einer alkoholbedingten Inzidenz von knapp 1,7 Mio. (95 % KI: 1.062.814–2.289.701) Fällen den größten Anteil aus, gefolgt von ca. 880.000 (95 % KI: 811.229–964.687) Fällen von Alkoholabhängigkeit, 240.000 (95 % KI: 51.907–426.130) Infektionskrankheiten, 51.500 (95 % KI: 48.592–55.860) Neuerkrankungen des Herz-Kreislauf-Systems, 25.700 (95 % KI: 20.955–30.615) Fällen von Leberzirrhose und Pankreatitis sowie 8250 (95 % KI: 6001–10.743) Fällen von Epilepsie. Eine Übersicht über Inzidenzen und Mortalität der berücksichtigten Erkrankungen und Verletzungen sind im Onlinematerial 2 verfügbar.

Die vermeidbaren inzidenten Erkrankungs- sowie Todesfälle für die jeweiligen Szenarien der Verbrauchssteuererhöhungen sind in Tab. 2 dargestellt (für eine vollständige Übersicht, siehe Onlinematerial 3). Entsprechend den 3 Szenarien hätten 40.150 (95 % KI: 33.305–48.749), 100.300 (95 % KI: 83.227–121.959) bzw. 200.400 (95 % KI: 166.228–244.150) neue alkoholbedingte Erkrankungs- und Verletzungsfälle sowie 560 (95 % KI: 472–701), 1400 (95 % KI: 1179–1742) bzw. 2800 (95 % KI: 2347–3471) Todesfälle vermieden werden können, wenn die derzeitigen Alkoholsteuern um jeweils 20 %, 50 % oder 100 % erhöht worden wären. Unter Berücksichtigung aller alkoholbedingten Erkrankungen sowie Verletzungen könnten durch eine Verdopplung der Verbrauchssteuern auf alkoholische Getränke in Deutschland schätzungsweise 6,9 % der alkoholbedingten inzidenten Krankheitsfälle und 6,7 % der Todesfälle vermieden werden (Tab. 2). Unter der Annahme, die Mehrkosten einer Steuererhöhung übertrugen sich vollständig auf die Konsumierenden, würde eine Verdopplung der Alkoholsteuern zu einer absoluten Preissteigerung von 10 Cent je Liter Bier und 5,21 € je Liter Spirituosen führen. Für Wein würde der Preis je Liter um 27 Cent steigen, wobei im Referenzszenario für Wein die gleiche Verbrauchssteuer wie für Bier angenommen wurde (siehe Methodik).

**Table 2:** 

	Vermeidbare Inzidenz		Vermeidbare Mortalität	
(%)	Anzahl	Anteil an alkoholbedingten Fällen (%)	Anzahl	Anteil an alkoholbedingten Fällen (%)
*Infektionskrankheiten*^a^				
20	2180 (477–3957)	0,91 (0,01–1,12)	–	–
50	5485 (1200–9970)	2,30 (0,02–2,82)	–	–
100	11.087 (2427–20.219)	4,65 (0,04–5,72)	–	–
*Erkrankungen des Herz-Kreislauf-Systems*^b^				
20	1544 (1280–1902)	3,00 (0,02–3,64)	265 (218–350)	2,17 (0,02–2,84)
50	3824 (3175–4694)	7,43 (0,06–8,96)	659 (542–864)	5,38 (0,04–7,04)
100	7526 (6263–9172)	14,62 (0,12–17,51)	1303 (1076–1707)	10,64 (0,08–13,74)
*Erkrankungen des Verdauungssystems*				
20	119 (93–286)	0,47 (0,00–1,01)	68 (56–88)	0,48 (0,00–0,63)
50	303 (236–716)	1,18 (0,01–2,54)	173 (142–224)	1,21 (0,01–1,61)
100	622 (485–1446)	2,42 (0,02–5,11)	355 (290–460)	2,49 (0,02–3,31)
*Alkoholabhängigkeit*				
20	22.043 (18.503–27.454)	2,50 (2,13–3,09)	154 (127–195)	2,42 (2,02–3,02)
50	54.860 (46.123–68.204)	6,22 (5,31–7,66)	384 (317–485)	6,02 (5,03–7,51)
100	108.848 (91.724–135.160)	12,34 (10,54–15,13)	763 (632–960)	11,96 (10,00–14,86)
*Epilepsie*				
20	71 (52–95)	0,86 (0,72–1,04)	5 (4–7)	0,87 (0,73–1,07)
50	179 (131–240)	2,17 (1,81–2,63)	13 (10–17)	2,20 (1,83–2,70)
100	362 (264–485)	4,39 (3,67–5,32)	26 (19–34)	4,45 (3,69–5,47)
*Unfälle und Verletzungen*				
20	14.193 (9876–17.893)	0,83 (0,01–1,03)	65 (46–84)	0,83 (0,01–1,02)
50	35.671 (24.790–45.053)	2,10 (0,02–2,60)	163 (117–213)	2,10 (0,02–2,56)
100	71.974 (49.922–91.198)	4,23 (0,04–5,23)	329 (235–430)	4,23 (0,03–5,18)
*Gesamt*				
20	40.150 (33.305–48.749)	1,38 (0,01–1,76)	557 (472–701)	1,35 (0,01–1,71)
50	100.322 (83.227–121.959)	3,45 (0,03–4,38)	1392 (1179–1742)	3,37 (0,03–4,27)
100	200.418 (166.228–244.150)	6,90 (0,06–8,72)	2776 (2347–3471)	6,73 (0,06–8,51)

95 % Konfidenzintervalle sind in Klammern angegeben

^a^ Für Infektionskrankheiten wurden ausschließlich vermeidbare, alkoholbedingte Krankheitsfälle modelliert

^b^ Für Erkrankungen des Herz-Kreislauf-Systems wurden ausschließlich vermeidbare, alkoholbedingte Todesfälle modelliert

Gemäß den Ergebnissen der Sensitivitätsanalyse, in der angenommen wurde, dass nur 80 % der Mehrkosten einer Steuererhöhung auf die Konsumierenden übertragen werden würden, hätten bei einer Verdopplung der Verbrauchssteuern bis zu 139.000 (95 % KI: 115.549–171.137) alkoholbedingte Erkrankungs- und Verletzungsfälle sowie 1900 (95 % KI: 1647–2398) Todesfälle vermieden werden können (siehe Onlinematerial 4). Dies entspricht 4,8 % (95 % KI: 0,0–5,9 %) der alkoholbedingten inzidenten Krankheitsfälle bzw. 4,7 % (95 % KI: 0,0–5,9 %) der alkoholbedingten Todesfälle.

**Figure 1:**
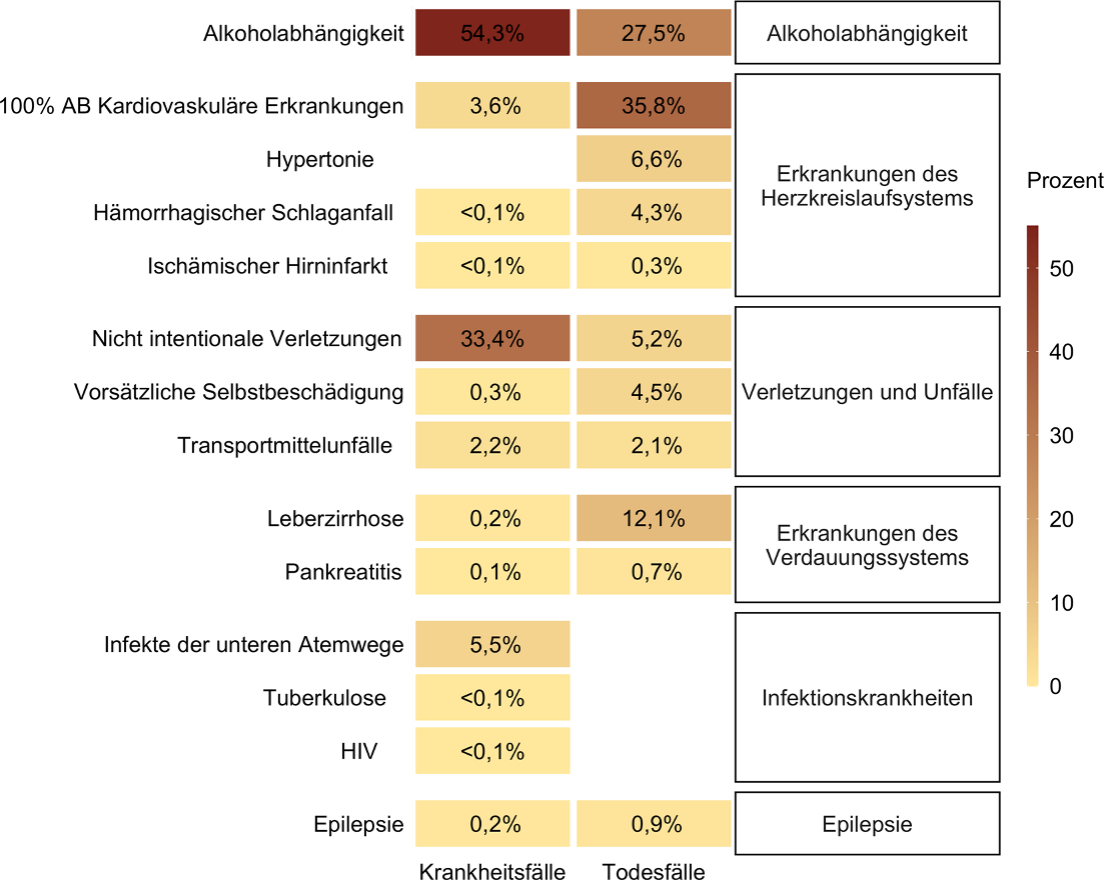

Abb. 1 veranschaulicht die prozentuale Verteilung der potenziell vermeidbaren Krankheits- sowie Todesfälle je Diagnose bei einer Verbrauchssteuererhöhung von 100 %. Alkoholabhängigkeit stellt den größten Anteil an potenziell vermeidbaren, inzidenten Krankheitsfällen dar (54,3 %). Alkoholbedingte Verletzungen und Unfälle waren für ca. ein Drittel der potenziell vermeidbaren Krankheitsfälle verantwortlich (35,9 %), wohingegen die verbleibenden Krankheitskategorien nur etwa einem Zehntel entsprachen. Die meisten Todesfälle hätten bei Erkrankungen des Herz-Kreislauf-Systems vermieden werden können (47,0 %), gefolgt von Alkoholabhängigkeit (27,5 %), Leberzirrhosen und Pankreatitis (12,8 %) sowie Verletzungen und Unfällen (11,8 %). Knapp 1 % der vermeidbaren Todesfälle ließen sich auf Epilepsie zurückführen.

## Diskussion

Der Konsum von Alkohol ist ein bedeutsamer veränderbarer Risikofaktor für die Krankheitslast in Deutschland. Die Ergebnisse unserer Modellierungsstudie zeigen, dass durch eine Verdoppelung der derzeitigen spezifischen Verbrauchssteuern auf Bier, Wein und Spirituosen die alkoholbedingte Krankheitslast in Deutschland substanziell reduziert werden könnte. Demnach hätten 2019 mehr als 200.000 oder 6,9 % der alkoholbedingten inzidenten Krankheits- und Verletzungsfälle sowie 2800 oder 6,7 % der alkoholbedingten Todesfälle vermieden werden können. Diese Zahlen berücksichtigen dabei nicht die potenziell vermiedenen Krebserkrankungen sowie Todesfälle (für Schätzungen vermeidbarer alkoholbedingter Krebserkrankungen in Deutschland, siehe [4, 40]) oder die durch Alkohol verursachten Todesfälle bei Infektionskrankheiten, die erst nach längerer Latenzzeit auftreten. Eine Verdopplung der derzeitigen getränkespezifischen Verbrauchssteuern, die einen bedeutenden Beitrag zur Reduzierung der alkoholbedingten Krankheitslast leisten kann, würde die Verkaufspreise für Bier um nur etwa 4 % und für Spirituosen um ca. 30 % erhöhen.

Die Ergebnisse unserer Modellierungsstudie veranschaulichen das bisher ungenutzte Potenzial erhöhter Verbrauchssteuern auf alkoholische Getränke in Deutschland für eine Reduzierung der alkoholbedingten Krankheitslast. Darüber hinaus spielen höhere Verbrauchssteuern ebenfalls eine wichtige Rolle für den Bundeshaushalt. Die jährlichen volkswirtschaftlichen Kosten des riskanten Alkoholkonsums in Deutschland werden auf ca. 39,3 Mrd. € geschätzt [41]. Den hohen ökonomischen sowie gesellschaftlichen Kosten von Alkohol steht dabei eine liberale Alkoholpolitik gegenüber, in der evidenzbasierte Empfehlungen der WHO zur Reduzierung der alkoholbedingten Krankheitslast nur unzureichend berücksichtigt werden. Erst 2020 veröffentlichte die WHO Empfehlungen zur Umsetzung effektiver Besteuerungen von Alkohol [11], von denen wesentliche Elemente derzeit keine Beachtung im deutschen Alkoholsteuersystem finden. Hierzu zählt unter anderem eine inflationsgebundene Besteuerung von Alkohol, wodurch bei steigender Inflation eine effektive Preiserhöhung aufgrund der Alkoholsteuern beibehalten wird (siehe [42]). Ferner sollten die Verbrauchssteuern auf dem Alkoholgehalt des jeweiligen alkoholischen Getränks basieren. In einer WHO-Publikation wurde geschätzt, dass in Deutschland ca. 19 % aller alkoholbedingten Todesfälle vermieden werden könnten, wenn es einen Mindeststeueranteil von 15 % auf den Einzelhandelspreis gäbe und der Preis pro Einheit Alkohol sich nicht nach Getränkeart unterscheiden würde [43]. In diesem Szenario würden insbesondere die Verbrauchssteuern auf Bier und Wein substanziell steigen, weshalb diese Schätzungen deutlich höher ausfallen als in unserer Studie.

Neben der Besteuerung von Alkohol gibt es weitere kosteneffektive Maßnahmen, die zu einer Reduktion des Alkoholkonsums sowie der alkoholbedingten Krankheitslast beitragen können. So kann beispielsweise ein Mindestpreis auf alkoholische Getränke bzw. ein Mindestpreis pro Einheit Alkohol zu einer bedeutsamen Reduktion des Pro-Kopf-Konsums sowie der alkoholbedingten Krankheitslast führen [11, 44]. Die Einführung eines Mindestpreises pro Einheit Alkohol (ca. 60 Cent pro 8 g Reinalkohol) in Schottland 2018 und Wales 2020 bedingte dort jeweils einen Rückgang von Alkoholkäufen um etwa 8 % im Jahr 2020 [45].

### Limitationen

Um die vermeidbare alkoholbedingte Krankheitslast in den verschiedenen Szenarien zu schätzen, wurden Annahmen zu den Modellparametern getroffen. Diese Annahmen ermöglichten die Modellierung potenziell vermeidbarer Krankheits- und Todesfälle, schränken allerdings die Interpretation und Generalisierbarkeit der Ergebnisse ein. Unser Modell berücksichtigt alters- und geschlechtsspezifische Informationen zum Alkoholkonsum und zur alkoholbedingten Krankheitslast, wohingegen die Verteilung der Getränkepräferenz sowie Preiselastizitäten über diese Gruppen hinweg als gleich angenommen wurden. Während eine kürzlich veröffentlichte Modellierungsstudie keine Hinweise auf substanzielle Geschlechterunterschiede in Bezug auf Preiselastizitäten fand [46], unterscheidet sich die Getränkepräferenz zwischen Frauen und Männern, was angesichts getränkespezifischer Preiselastizitäten wiederum Auswirkungen auf den geschätzten verringerten Alkoholkonsum und damit auf die resultierende vermeidbare Krankheitsinzidenz sowie Mortalität haben kann. Weiterhin wurden sogenannte Kreuzelastizitäten zwischen alkoholischen Getränken sowie zwischen diesen und anderen Substanzen nicht berücksichtigt, da aus den vorliegenden Studien nicht eindeutig erkennbar ist, inwiefern eine Preiserhöhung eines Getränks mit der Veränderung der Konsumnachfrage anderer Getränke oder Substanzen einhergeht [47].

Eine weitere Annahme war, dass die Veränderungen der Verbrauchssteuern vollständig auf die Konsumierenden übertragen werden. Darüber hinaus wurden nur 3 Getränketypen modelliert und Schaumweine, die in Deutschland einer gesonderten Verbrauchssteuer unterliegen [17], nicht getrennt von stillen Weinen berücksichtigt. Die Qualität der in der Modellierung berücksichtigten Alkoholkonsumdaten schätzen wir insgesamt als gut ein, da der registrierte Alkoholkonsum größtenteils durch Steuerdaten ermittelt wird [1]. Lediglich die Berücksichtigung von geschlechts- und altersspezifischen Daten des Alkoholkonsums kann zu Verzerrungen führen, da diese mittels Umfragedaten geschätzt werden, welche den Pro-Kopf-Konsum in der Regel substanziell unterschätzen [48]. Dies würde die Ergebnisse jedoch nur dann beeinflussen, wenn diese Untererfassung systematisch zwischen den Alters- und Geschlechtergruppen variieren würde.

## Fazit

In kaum einem anderen europäischen Land wird so viel Alkohol konsumiert wie in Deutschland, was zu einer erheblichen alkoholbedingten Krankheitslast führt. Unsere Modellierungsstudie hat gezeigt, dass kurzfristig einer von 15 alkoholbedingten Erkrankungs- sowie Todesfällen potenziell vermieden werden könnte, wenn die derzeitigen Verbrauchssteuern auf alkoholische Getränke verdoppelt würden. Alkoholbedingte Erkrankungen sind vermeidbar und eine sinnvolle Besteuerung alkoholischer Getränke erscheint als eine hierfür geeignete Maßnahme, wie unsere sowie weitere Studien gezeigt haben [4, 10, 12, 21, 40, 43, 49]. Verbrauchssteuern auf alkoholische Getränke sollten als Gesundheitssteuer verstanden werden und ihre Etablierung insbesondere in Deutschland eine Priorität für die öffentliche Gesundheit sein.

## Supplementary Information





## References

[1] Manthey J, Shield K, Rylett M, Hasan O, Probst C, Rehm J (2019). Alcohol exposure between 1990 and 2017 and forecasts until 2030: a global modelling study. Lancet.

[2] Rehm J, Gmel GE, Gmel G, Hasan OSM, Imtiaz S, Popova S, Probst C, Roerecke M, Room R, Samokhvalov AV, Shield KD, Shuper PA (2017). The relationship between different dimensions of alcohol use and the burden of disease—an update. Addiction.

[3] GBD 2019 Risk Factors Collaborators (2020). Global burden of 87 risk factors in 204 countries and territories, 1990–2019: a systematic analysis for the Global Burden of Disease Study 2019. Lancet.

[4] Gredner T, Niedermaier T, Brenner H, Mons U (2021). Impact of reducing alcohol consumption through price-based policies on cancer incidence in Germany 2020 to 2050—a simulation study. Addiction.

[5] World Health Organization (2013). Global action plan for the prevention and control of noncommunicable diseases 2013–2020.

[6] United Nations (2015). Transforming our world: the 2030 agenda for sustainable development.

[7] World Health Organization (2017). Tackling NCDs: “best buys” and other recommended interventions for the prevention and control of noncommunicable diseases.

[8] World Health Organization (2018). Global status report on alcohol and health 2018.

[9] World Health Organization (2021). Making the WHO European Region SAFER: developments in alcohol control policies, 2010–2019.

[10] Chisholm D, Moro D, Bertram M, Pretorius C, Gmel G, Shield K, Rehm J (2018). Are the “best buys” for alcohol control still valid? An update on the comparative cost-effectiveness of alcohol control strategies at the global level. J Stud Alcohol Drugs.

[11] World Health Organization (2020). Alcohol pricing in the WHO European Region. Update report on the evidence and recommended policy actions.

[12] Stockwell T, Churchill S, Sherk A, Sorge J, Gruenewald P (2020). How many alcohol-attributable deaths and hospital admissions could be prevented by alternative pricing and taxation policies? Modelling impacts on alcohol consumption, revenues and related harms in Canada. Health Promot Chronic Dis Prev Can.

[13] Statista (2020) Alcoholic drinks—price per unit. Europe. [Data set]. https://www.statista.com/outlook/10000000/102/alcoholic-drinks/europe. Zugegriffen: 31. Juli 2020

[14] European Commission (2020). Excise duty on alcohol.

[15] Bundesministerium der Justiz und für Verbraucherschutz (2013). Alkoholsteuergesetz.

[16] Bundesministerium der Justiz und für Verbraucherschutz (2009). Biersteuergesetz.

[17] Bundesministerium der Justiz und für Verbraucherschutz (2009). Schaumwein- und Zwischenerzeugnissteuergesetz (SchaumwZwStG).

[18] Bundesministerium der Justiz und für Verbraucherschutz (2004). Gesetz über die Erhebung einer Sondersteuer auf alkoholhaltige Süßgetränke (Alkopops) zum Schutz junger Menschen (Alkopopsteuergesetz – AlkopopStG).

[19] Angus C, Holmes J, Meier P (2019). Comparing alcohol taxation throughout the European Union. Addiction.

[20] Manthey J, Kilian C, Carr S, Rehm J, Stöver H, Werse B, Kluge Haberkorn C (2021). Besteuerung von Alkohol in Deutschland. 8. Alternativer Drogen- und Suchtbericht 2021.

[21] Rovira P, Kilian C, Neufeld M, Rumgay H, Soerjomataram I, Ferreira-Borges C, Shield KD, Sornpaisarn B, Rehm J (2021). Fewer cancer cases in four countries of the WHO European Region in 2018 through increased alcohol excise taxation: a modelling study. Eur Addict Res.

[22] Wagenaar AC, Maldonado-Molina MM, Wagenaar BH (2009). Effects of alcohol tax increases on alcohol-related disease mortality in alaska: time-series analyses from 1976 to 2004. Am J Public Health.

[23] Grundy A, Poirier AE, Khandwala F, McFadden A, Friedenreich CM, Brenner DR (2016). Cancer incidence attributable to alcohol consumption in Alberta in 2012. CMAJ Open.

[24] Zeisser C, Stockwell TR, Chikritzhs T (2014). Methodological biases in estimating the relationship between alcohol consumption and breast cancer: the role of drinker misclassification errors in Meta-analytic results. Alcohol Clin Exp Res.

[25] Morojele NK, Shenoi SV, Shuper PA, Braithwaite RS, Rehm J (2021). Alcohol use and the risk of communicable diseases. Nutrients.

[26] Global Health Data Exchange (GHDx) (2021). GBD results tool for the global burden of disease 2017 study.

[27] World Health Organization (2021). WHO global information system on alcohol and health (GISAH).

[28] Bundesverband der Deutschen Spirituosen-Industrie und -Importeure e. V. (2021). Daten aus der Alkoholwirtschaft 2021.

[29] Statistisches Bundesamt (2021). Verbraucherpreisindex: Deutschland, Jahre, Klassifikation der Verwendungszwecke des Individualkonsums – Bier, Wein und Spirituosen.

[30] Deutsches Weininstitut (2020). Deutsche Wein Statistik 2019/2020.

[31] Sornpaisarn B, Shield KD, Österberg E, Rehm J (2017). Resource tool on alcohol taxation and pricing policies.

[32] Ornstein SI, Levy D, Galanter M, Begleiter H, Cicero T, Deitrich R, Goodwin DW (1983). Price and income elasticities and the demand for alcoholic beverages. Recent developments in alcoholism.

[33] Fogarty J (2010). The demand of beer, wine and spirits: a survey of the literature. J Econ Surv.

[34] American Psychiatric Association (1994). Diagnostic and statistical manual of mental disorders DSM-IV.

[35] Wagenaar AC, Salois MJ, Komro KA (2009). Effects of beverage alcohol price and tax levels on drinking: a meta-analysis of 1003 estimates from 112 studies. Addiction.

[36] Shield KD, Manthey J, Rylett M, Probst C, Wettlaufer A, Parry CDH, Rehm J (2020). National, regional, and global burdens of disease from 2000 to 2016 attributable to alcohol use: a comparative risk assessment study. Lancet Public Health.

[37] Stockwell T, Zhao J, Sherk A, Rehm J, Shield K, Naimi T (2018). Underestimation of alcohol consumption in cohort studies and implications for alcohol’s contribution to the global burden of disease: Underestimation of alcohol consumption. Addiction.

[38] Churchill S, Angus C, Purshouse R, Brennan A, Sherk A (2020). Expanding attributable fraction applications to outcomes wholly attributable to a risk factor. Stat Methods Med Res.

[39] Gmel G, Shield KD, Frick H, Kehoe T, Gmel G, Rehm J (2011). Estimating uncertainty of alcohol-attributable fractions for infectious and chronic diseases. BMC Med Res Methodol.

[40] Kilian C, Rovira P, Neufeld M, Ferreira-Borges C, Rumgay H, Soerjomataram I, Rehm J (2021). Modelling the impact of increased alcohol taxation on alcohol-attributable cancers in the WHO European Region. Lancet Reg Health Eur.

[41] Effertz T, Verheyen F, Linder R (2017). The costs of hazardous alcohol consumption in Germany. Eur J Health Econ.

[42] Voon D, Fogarty J (2021). Role of alcohol taxes in moderating alcohol consumption: current and future potential impacts. Drug Alcohol Rev.

[43] Neufeld M, Rovira P, Ferreira-Borges C, Kilian C, Sassi F, Veryga A, Rehm J (2022). Impact of introducing a minimum alcohol tax share in retail prices on alcohol-attributable mortality in the WHO European region: a modelling study. Lancet Reg Health Eur.

[44] Boniface S, Scannell JW, Marlow S (2017). Evidence for the effectiveness of minimum pricing of alcohol: a systematic review and assessment using the Bradford Hill criteria for causality. BMJ Open.

[45] Anderson P, O’Donnell A, Kaner E, Llopis EJ, Manthey J, Rehm J (2021). Impact of minimum unit pricing on alcohol purchases in Scotland and Wales: controlled interrupted time series analyses. Lancet Public Health.

[46] Meier PS, Holmes J, Brennan A, Angus C (2021). Alcohol policy and gender: a modelling study estimating gender-specific effects of alcohol pricing policies. Addiction.

[47] Meng Y, Brennan A, Purshouse R, Hill-McManus D, Angus C, Holmes J, Meier PS (2014). Estimation of own and cross price elasticities of alcohol demand in the UK—a pseudo-panel approach using the Living Costs and Food Survey 2001–2009. J Health Econ.

[48] Kilian C, Manthey J, Probst C, Brunborg GS, Bye EK, Ekholm O, Kraus L, Moskalewicz J, Sierosławski J, Rehm J (2020). Why is per capita consumption underestimated in alcohol surveys? Results from 39 surveys in 23 European countries. Alcohol Alcohol.

[49] Saar I (2015). Do Alcohol Excise Taxes Affect Traffic Accidents? Evidence From Estonia. Traffic Inj Prev.

