# Supplementary material for: Potential impact of increased alcohol taxes on the alcohol-attributable burden of disease in Germany: a modelling study
Source: Bundesgesundheitsblatt Gesundheitsforschung Gesundheitsschutz. 2022 Apr 19;65(6):668–76. [Article in German] doi: 10.1007/s00103-022-03528-9 (PMC9132832; doi:10.1007/s00103-022-03528-9)
Supplement: Supplementary file 1 [file 103_2022_3528_MOESM1_ESM.docx]

**Bundesgesundheitsblatt – Gesundheitsforschung – Gesundheitsschutz, Ausgabe 6/2022**

Onlinematerial zum Beitrag:

**Potenzielle Auswirkungen erhöhter Alkoholsteuern auf die alkoholbedingte Krankheitslast in Deutschland: Eine Modellierungsstudie**

Autor*innen: Carolin Kilian, Pol Rovira, Maria Neufeld, Jakob Manthey & Jürgen Rehm

Korrespondenzadresse:

Dr. Carolin Kilian

Chemnitzer Straße 46

01187 Dresden

carolin.kilian@tu-dresden.de

# **Übersicht:**

[Onlinematerial 1: Schätzung der Krankheitslast sowie vermeidbarer Krankheits- und Todesfälle für 100% alkoholbedingte Erkrankungen 2](#_Toc89942323)

[Onlinematerial 2: Inzidenzen und Mortalität für Deutschland im Jahr 2019 3](#_Toc89942324)

[Onlinematerial 3: Vermeidbare alkoholbedingte Krankheits- und Verletzungsfälle sowie Todesfälle nach Krankheitsdiagnose 5](#_Toc89942325)

[Onlinematerial 4: Sensitivitätsanalyse 7](#_Toc89942326)

# **Onlinematerial 1: Schätzung der Krankheitslast sowie vermeidbarer Krankheits- und Todesfälle für 100% alkoholbedingte Erkrankungen**

Um die Krankheitsinzidenz bzw. Mortalität in den verschiedenen Szenarien für Erkrankungen zu schätzen, die vollständig auf den Konsum von Alkohol zurückzuführen sind, wurde die Methodik von Churchill et al. angewendet [38]. Für 100% alkoholbedingte Erkrankungen wurde demnach eine Risikofunktion angenommen, die exponentiell mit dem konsumierten Alkohol ansteigt und lediglich bei starkem Alkoholkonsum (40 Gramm Reinalkohol pro Tag für Frauen und 60 Gramm Reinalkohol pro Tag für Männer) berücksichtigt wurde. Die Funktion ist in Gleichung 1 dargestellt, wobei *t* der Schwellenwert für starken Alkoholkonsum darstellt.

*Gleichung 1:* $p\left( x;k,t \right)=\left\{ \begin{aligned} 0 x<t \\ \exp\left( k\left( x-t \right) \right)-1 x\geq t \end{aligned} \right.$

Weiterhin wird angenommen, dass das Integral der Funktion über der Gamma-Verteilung gleich der Anzahl der Erkrankungen (*N*) über die gesamte Population (*S*) ist; siehe Gleichung 2, wobei $df\left( x;\mu\right)$ die Gamma-Verteilung der Population mit einem mittleren Konsum $\mu$ ist.

*Gleichung 2:* $\int_{0}^{150} p\left( x;k,t \right) df\left( x;\mu\right)=\frac{N}{S}$

Die Inzidenz bzw. Mortalität der jeweiligen Erkrankung unter jedem Szenario wird mittels Gleichung 2 bestimmt, die für jede Erkrankung *k* sowie für Geschlecht und Alter angepasst wird.

Im Fall der Alkoholabhängigkeit wurden Vergiftungen durch Alkohol zur Schätzung der vermeidbaren Todesfälle berücksichtigt.

# **Onlinematerial 2: Inzidenzen und Mortalität für Deutschland im Jahr 2019**

**Tabelle S1. Alkoholbezogene sowie alkoholbedingte Inzidenz von Erkrankungen sowie Verletzungen im Jahr 2019.**

| **Krankheit/Verletzung** | **Alkoholbezogene Inzidenz^a^** | **Alkoholbedingte Inzidenz^b^** | |  |
| --- | --- | --- | --- | --- |
| **Infektionskrankheiten** | | |  |  |
| Tuberkulose | 4.706 (4.258-5.149) | 1.775 (497-3.086) | |  |
| Humane Immundefizienz-Viruskrankheit | 2.300 (1.917-2.674) | 117 (81-158) | |  |
| Infekte der unteren Atemwege | 2.780.715 (2.657.480-2.903.087) | 236.747 (50.911-424.605) | |  |
| **Erkrankungen des Herzkreislaufsystems** | | | |  |
| Ischämischer Hirninfarkt | 98.726 (89.777-107.960) | 124 (0-2.913) | |  |
| Hämorrhagischer Schlaganfall | 18.803 (17.516-20.069) | 2.736 (1.947-3.597) | |  |
| 100% AB kardiovaskuläre Erkrankungen (Endokarditis, Kardiomyopathie und Myokarditis) | 48.626 (45.654-51.578) | 48.626 (45.654-51.578) | |  |
| **Erkrankungen des Verdauungssystems** | | |  |  |
| Leberzirrhose | 24.981 (21.381-28.735) | 17.764 (14.740-20.756) | |  |
| Pankreatitis | 36.996 (34.044-39.906) | 7.888 (4.389-12.010) | |  |
| **Alkoholabhängigkeit** (100% AB) | 882.185 (811.229-964.687) | 882.185 (811.229-964.687) | |  |
| **Epilepsie** | 35.770 (30.716-41.190) | 8.256 (6.001-10.743) | |  |
| **Unfälle und Verletzungen** | | | |  |
| Transportmittelunfälle | 312.598 (286.467-337.059) | 127.566 (78.953-170.365) | |  |
| Nicht intentionale Verletzungen | 6.612.319 (6.313.871-6.939.571) | 1.557.090 (938.328-2.130.924) | |  |
| Vorsätzliche Selbstbeschädigung | 61.021 (58.244-63.980) | 15.552 (9.498-21.472) | |  |
| **Gesamt** | 10.919.744 (10.574.732-11.274.436) | 2.906.428 (2.228.037-3.537.265) | |  |

100% AB: Erkrankungen sind zu 100% alkoholbedingt, d. h. auf den Konsum von Alkohol zurückführbar. 95% Konfidenzintervalle sind in Klammern angegeben. Referenz: [26].

^a^ inzidente Erkrankungsfälle, die vollständig oder teilweise durch Alkoholkonsum verursacht sind

^b^ inzidente Erkrankungsfälle, die auf den Konsum von Alkohol zurückzuführen sind

**Tabelle S2. Alkoholbezogene sowie alkoholbedingte Mortalität von Erkrankungen sowie Verletzungen im Jahr 2019.**

| **Krankheit/Verletzung** | **Alkoholbezogene Mortalität^a^** | **Alkoholbedingte Mortalität^b^** | |  |
| --- | --- | --- | --- | --- |
| **Erkrankungen des Herzkreislaufsystems** | | | |  |
| Ischämischer Hirninfarkt | 51.674 (46.635-56.492) | 11 (0-1.481) | |  |
| Hämorrhagischer Schlaganfall | 15.530 (14.086-16.892) | 2.196 (1.515-2.908) | |  |
| Hypertonie | 34.349 (28.300-40.548) | 3.100 (2.229-4.195) | |  |
| 100% AB kardiovaskuläre Erkrankungen (Endokarditis, Kardiomyopathie und Myokarditis) | 6.941 (6.234-7.573) | 6.941 (6.234-7.573) | |  |
| **Erkrankungen des Verdauungssystems** | | |  |  |
| Leberzirrhose | 20.488 (19.524-21.566) | 13.692 (12.205-14.979) | |  |
| Pankreatitis | 2.173 (1.903-2.446) | 565 (325-848) | |  |
| **Alkoholabhängigkeit** (100% AB) | 6.379 (5.904-6.854) | 6.379 (5.904-6.854) | |  |
| **Epilepsie** | 2.671 (2.315-3.018) | 581 (424-728) | |  |
| **Unfälle und Verletzungen** | | | |  |
| Transportmittelunfälle | 4.380 (4.190-4.573) | 1.683 (1.023-2.288) | |  |
| Nicht intentionale Verletzungen | 18.674 (17.230-20.081) | 3.010 (1.744-4.434) | |  |
| Vorsätzliche Selbstbeschädigung | 11.645 (11.112-12.107) | 3.078 (1.834-4.337) | |  |
| **Gesamt** | 174.903 (166.422-183.390) | 41.235 (38.125-45.085) | |  |

100% AB: Erkrankungen sind zu 100% alkoholbedingt, d. h. auf den Konsum von Alkohol zurückführbar. 95% Konfidenzintervalle sind in Klammern angegeben. Referenz: [26].

^a^ Todesfälle, die vollständig oder teilweise durch Alkoholkonsum verursacht sind

^b^ Todesfälle, die auf den Konsum von Alkohol zurückzuführen sind

# **Onlinematerial 3: Vermeidbare alkoholbedingte Krankheits- und Verletzungsfälle sowie Todesfälle nach Krankheitsdiagnose**

**Tabelle S3. Vermeidbare alkoholbedingte Krankheits- und Verletzungsfälle im Jahr 2019 für die drei Szenarien einer Verbrauchssteuererhöhung für alkoholische Getränke.**

| **Krankheit/Verletzung** | **Szenario 1: 20%** | | **Szenario 2: 50%** | **Szenario 3: 100%** |
| --- | --- | --- | --- | --- |
| **Infektionskrankheiten** | | | | |
| Tuberkulose | 14 (4-20) | | 35 (11-52) | 71 (23-105) |
| Humane Immundefizienz-Viruskrankheit | 2 (1-3) | | 5 (3-6) | 10 (7-13) |
| Infekte der unteren Atemwege | 2.165 (467-3.941) | | 5.446 (1.175-9.925) | 11.006 (2.374-20.138) |
| **Erkrankungen des Herzkreislaufsystems** | | | | |
| Ischämischer Hirninfarkt | 18 (-2-87) | | 46 (-7-216) | 93 (-17-429) |
| Hämorrhagischer Schlaganfall | 30 (19-43) | | 74 (47-108) | 149 (95-216) |
| 100% AB kardiovaskuläre Erkrankungen (Endokarditis, Kardiomyopathie und Myokarditis) | 1.496 (1.246-1.839) | | 3.704 (3.092-4.542) | 7.283 (6.102-8.895) |
| **Erkrankungen des Verdauungssystems** | | | | |
| Leberzirrhose | 69 (53-95) | | 176 (135-242) | 362 (278-499) |
| Pankreatitis | 50 (33-214) | | 127 (82-535) | 260 (167-1.071) |
| **Alkoholabhängigkeit** (100% AB) | 22.043 (18.503-27.454) | | 54.860 (46.123-68.204) | 108.848 (91.724-135.160) |
| **Epilepsie** | | 71 (52-95) | 179 (131-240) | 362 (264-485) |
| **Unfälle und Verletzungen** | | | |  |
| Transportmittelunfälle | 864 (650-1.048) | | 2.177 (1.635-2.652) | 4.412 (3.299-5.401) |
| Nicht intentionale Verletzungen | 13.196 (8.971-16.792) | | 33.161 (22.520-42.285) | 66.891 (45.354-85.596) |
| Vorsätzliche Selbstbeschädigung | 132 (91-169) | | 333 (228-424) | 671 (459-858) |
| **Gesamt** | 40.150 (33.305-48.749) | | 100.322 (83.227-121.959) | 200.418 (166.228-244.150) |

100% AB: Erkrankungen sind zu 100% alkoholbedingt, d. h. auf den Konsum von Alkohol zurückführbar. 95% Konfidenzintervalle sind in Klammern angegeben.

**Tabelle S4. Vermeidbare alkoholbedingte Todesfälle im Jahr 2019 für die drei Szenarien einer Verbrauchssteuererhöhung für alkoholische Getränke.**

| **Krankheit/Verletzung** | **Szenario 1: 20%** | **Szenario 2: 50%** | **Szenario 3: 100%** |
| --- | --- | --- | --- |
| **Erkrankungen des Herzkreislaufsystems** | | | |
| Ischämischer Hirninfarkt | 2 (-6-52) | 4 (-16-132) | 8 (-34-258) |
| Hämorrhagischer Schlaganfall | 24 (15-35) | 60 (39-88) | 120 (77-177) |
| Hypertonie | 36 (20-61) | 91 (51-151) | 182 (101-302) |
| 100% AB kardiovaskuläre Erkrankungen (Endokarditis, Kardiomyopathie und Myokarditis) | 203 (162-257) | 504 (403-635) | 993 (797-1247) |
| **Erkrankungen des Verdauungssystems** | | | |
| Leberzirrhose | 64 (52-81) | 163 (132-207) | 335 (270-427) |
| Pankreatitis | 4 (2-11) | 10 (6-27) | 20 (12-52) |
| **Alkoholabhängigkeit** (100% AB) | 154 (127-195) | 384 (317-485) | 763 (632-960) |
| **Epilepsie** | 5 (4-7) | 13 (10-17) | 26 (19-34) |
| **Unfälle und Verletzungen** | | |  |
| Transportmittelunfälle | 11 (9-14) | 29 (22-35) | 59 (44-71) |
| Nicht intentionale Verletzungen | 29 (18-40) | 72 (46-101) | 144 (92-203) |
| Vorsätzliche Selbstbeschädigung | 25 (17-32) | 62 (44-81) | 126 (88-165) |
| **Gesamt** | 557 (472-701) | 1.392 (1.179-1.742) | 2.776 (2.347-3.471) |

100% AB: Erkrankungen sind zu 100% alkoholbedingt, d. h. auf den Konsum von Alkohol zurückführbar. 95% Konfidenzintervalle sind in Klammern angegeben.

# **Onlinematerial 4: Sensitivitätsanalyse**

**Tabelle S5. Sensitivitätsanalyse: Vermeidbare alkoholbedingte Krankheits- und Verletzungsfälle im Jahr 2019 für die drei Szenarien einer Verbrauchssteuererhöhung für alkoholische Getränke; nur 80% der steuerlichen Preiserhöhung werden auf die Verbraucher*innen übertragen.**

| **Krankheit/Verletzung** | | **Szenario 1: 20%** | **Szenario 2: 50%** | **Szenario 3: 100%** |
| --- | --- | --- | --- | --- |
| **Infektionskrankheiten** | | | | |
| Tuberkulose | | 4 (1-6) | 20 (6-29) | 49 (15-70) |
| Humane Immundefizienz-Viruskrankheit | | 1 (0-1) | 3 (2-4) | 7 (5-9) |
| Infekte der unteren Atemwege | | 575 (109-1.154) | 3.180 (604-6.003) | 7.582 (1.437-14.100) |
| **Erkrankungen des Herzkreislaufsystems** | | | | |
| Ischämischer Hirninfarkt | | 5 (-1-25) | 27 (-3-134) | 64 (-8-305) |
| Hämorrhagischer Schlaganfall | | 8 (5-12) | 43 (27-62) | 103 (65-148) |
| 100% AB kardiovaskuläre Erkrankungen (Endokarditis, Kardiomyopathie und Myokarditis) | | 392 (261-543) | 2.179 (1851-2.626) | 5.095 (4.311-6.115) |
| **Erkrankungen des Verdauungssystems** | | | | |
| Leberzirrhose | | 18 (12-26) | 102 (81-135) | 247 (193-328) |
| Pankreatitis | | 14 (9-58) | 74 (53-318) | 178 (127-742) |
| **Alkoholabhängigkeit** (100% AB) | | 5.866 (4.132-8.061) | 32.263 (27.430-39.177) | 75.827 (64.070-93.064) |
| **Epilepsie** | 19 (12-28) | | 104 (76-138) | 249 (182-332) |
| **Unfälle und Verletzungen** | | | |  |
| Transportmittelunfälle | | 229 (146-312) | 1.270 (962-1.521) | 3.034 (2.257-3.664) |
| Nicht intentionale Verletzungen | | 3.501 (2.152-5.066) | 19.376 (12.828-25.336) | 46.127 (30.609-61.030) |
| Vorsätzliche Selbstbeschädigung | | 35 (21-50) | 194 (128-253) | 463 (300-605) |
| **Gesamt** | | 10.666 (7.437-14.607) | 58.838 (49.354-71.082) | 139.024 (115.549-171.137) |

100% AB: Erkrankungen sind zu 100% alkoholbedingt, d. h. auf den Konsum von Alkohol zurückführbar. 95% Konfidenzintervalle sind in Klammern angegeben.

**Tabelle S6. Sensitivitätsanalyse: Vermeidbare alkoholbedingte Todesfälle im Jahr 2019 für die drei Szenarien einer Verbrauchssteuererhöhung für alkoholische Getränke; nur 80% der steuerlichen Preiserhöhung werden auf die Verbraucher*innen übertragen.**

| **Krankheit/Verletzung** | **Szenario 1: 20%** | **Szenario 2: 50%** | **Szenario 3: 100%** |
| --- | --- | --- | --- |
| **Erkrankungen des Herzkreislaufsystems** | | | |
| Ischämischer Hirninfarkt | 0 (-1-14) | 2 (-5-73) | 6 (-12-170) |
| Hämorrhagischer Schlaganfall | 6 (4-10) | 35 (23-51) | 83 (53-122) |
| Hypertonie | 9 (5-17) | 53 (31-93) | 126 (72-221) |
| 100% AB kardiovaskuläre Erkrankungen (Endokarditis, Kardiomyopathie und Myokarditis) | 53 (36-75) | 296 (243-370) | 694 (564-872) |
| **Erkrankungen des Verdauungssystems** | | | |
| Leberzirrhose | 17 (11-23) | 95 (78-118) | 229 (187-288) |
| Pankreatitis | 1 (1-3) | 6 (4-16) | 13 (9-37) |
| **Alkoholabhängigkeit** (100% AB) | 41 (28-57) | 226 (190-279) | 531 (442-664) |
| **Epilepsie** | 1 (1-2) | 7 (5-10) | 18 (13-23) |
| **Unfälle und Verletzungen** | | |  |
| Transportmittelunfälle | 3 (2-4) | 17 (13-20) | 40 (30-49) |
| Nicht intentionale Verletzungen | 8 (4-12) | 42 (25-59) | 100 (60-141) |
| Vorsätzliche Selbstbeschädigung | 7 (4-10) | 37 (24-48) | 87 (57-114) |
| **Gesamt** | 147 (102-203) | 816 (706-1.015) | 1.926 (1.647-2.398) |

100% AB: Erkrankungen sind zu 100% alkoholbedingt, d. h. auf den Konsum von Alkohol zurückführbar. 95% Konfidenzintervalle sind in Klammern angegeben.
